# scHiCEmbed: Bin-Specific Embeddings of Single-Cell Hi-C Data Using Graph Auto-Encoders

**DOI:** 10.3390/genes13061048

**Published:** 2022-06-11

**Authors:** Tong Liu, Zheng Wang

**Affiliations:** Department of Computer Science, University of Miami, 1365 Memorial Drive, P.O. Box 248154, Coral Gables, FL 33124, USA; tong.liu@miami.edu

**Keywords:** single-cell Hi-C data, embedding, graph auto-encoders, 3D-genome-structure reconstruction, TAD detection, cell-type clustering

## Abstract

Most publicly accessible single-cell Hi-C data are sparse and cannot reach a higher resolution. Therefore, learning latent representations (bin-specific embeddings) of sparse single-cell Hi-C matrices would provide us with a novel way of mining valuable information hidden in the limited number of single-cell Hi-C contacts. We present scHiCEmbed, an unsupervised computational method for learning bin-specific embeddings of single-cell Hi-C data, and the computational system is applied to the tasks of 3D structure reconstruction of whole genomes and detection of topologically associating domains (TAD). The only input of scHiCEmbed is a raw or scHiCluster-imputed single-cell Hi-C matrix. The main process of scHiCEmbed is to embed each node/bin in a higher dimensional space using graph auto-encoders. The learned n-by-3 bin-specific embedding/latent matrix is considered the final reconstructed 3D genome structure. For TAD detection, we use constrained hierarchical clustering on the latent matrix to classify bins: *S_Dbw* is used to determine the optimal number of clusters, and each cluster is considered as one potential TAD. Our reconstructed 3D structures for individual chromatins at different cell stages reveal the expanding process of chromatins during the cell cycle. We observe that the TADs called from single-cell Hi-C data are not shared across individual cells and that the TAD boundaries called from raw or imputed single-cell Hi-C are significantly different from those called from bulk Hi-C, confirming the cell-to-cell variability in terms of TAD definitions. The source code for scHiCEmbed is publicly available, and the URL can be found in the conclusion section.

## 1. Introduction

The Hi-C techniques [1] provide a powerful way of exploring the three-dimensional (3D) organization of whole genomes; it can capture genome-wide contacts, which allows us to generate the two-dimensional (2D) contact maps of whole genomes. In the last eleven years, there have been lots of variants of the original Hi-C experiment, such as in situ Hi-C [2], DNase Hi-C [3], Micro-C [4], HiChiP [5], and Capture Hi-C [6]. Hi-C and its variants play a vital role in discovering A/B compartments [1], topologically associating domains (TADs) [7], and chromatin loops [2]. They also have been used to reconstruct 3D genome structures [8,9,10], predict DNA methylation [11], and detect structural genome variations [12,13]. Since the original Hi-C and its variants collect billions of contacts from a population of cells (usually about a million cells but at least ten thousand cells), the corresponding population/bulk Hi-C contact maps represent an ensemble of respective organizations of these millions of nuclei [2,12]. Therefore, bulk Hi-C cannot efficiently represent underlying cell-to-cell variances.

In 2013, the single-cell Hi-C technique was present to capture the DNA proximities of individual cells [14], which firstly reveal cell-to-cell variability in chromosomal conformation. Since then, single-cell Hi-C experiments have been conducted on different cell types [15,16,17,18,19,20,21]. Ramani et al. [17] conducted single-cell combinatorial indexed Hi-C (sciHi-C) experiments, which can simultaneously generate the single-cell Hi-C contact maps for thousands of cells. The sciHi-C experiment has generated 10,696 single-cell contact maps, and they also found cell-to-cell variance between these single-cell Hi-C contact maps. Recently, single-cell DNA methylation and Hi-C contacts can be captured simultaneously [19,21]. The 3D genome structures of individual cells can be reconstructed using single-cell Hi-C data [16,22]. 

TADs are a self-interacting genomic region [7]. The Hi-C contacts within a TAD are more enriched than the contacts between two successive TADs. Researchers [7] also found that the boundaries of TADs are enriched with some histone modifications and CCCTC-binding factor (CTCF), which plays a weaver role in mediating intra- and inter-chromosomal contacts [23]. TADs detected in bulk Hi-C have been well studied [2,7,24], and there are lots of computational methods for detecting TADs in bulk Hi-C [25,26,27,28,29,30]. However, detecting TADs in single-cell Hi-C data using these computational methods may not be successful because there are a lot of isolated and missing contacts in single-cell Hi-C matrices, which make TAD boundaries unclear. Too many isolated contacts around the diagonal of single-cell Hi-C matrices at a higher resolution would weaken the efficiency of the two widely used methods: directionality index (DI) [7] and insulation score [31]. 

Imputation and embedding methods can be used to deal with the challenges caused by the isolated and missing contacts in the single-cell Hi-C matrices. The scHiCluster [32] is an imputation method for single-cell Hi-C data for the task of cell type clustering. The main procedure of scHiCluster is a two-stage imputation process: linear convolution and random walk. It has been shown that scHiCluster-imputed single-cell Hi-C matrices lead to better cell-type clustering than raw single-cell Hi-C data. Another potential method for enriching contacts in single-cell Hi-C matrices would be node embedding for graph-structured data [33], in which we can do link prediction for interpolating missing contacts, bin-specific embeddings for 3D genome reconstruction, and node clustering for detecting TADs. 

In this work, we present scHiCEmbed, an unsupervised computational method based on graph auto-encoders to learn node embeddings from single-cell Hi-C data. The input of scHiCEmbed is a raw or scHiCluster-imputed single-cell Hi-C matrix, which is taken as an adjacency matrix of graph-structured data. The learning process is to interpret latent representations of single-cell Hi-C matrices, which are used to recover the input matrix. The learned node embedding matrix is further used to reconstruct 3D genomes and detect TADs.

## 2. Materials and Methods

### 2.1. Hi-C Data Processing

We used four single-cell Hi-C data sets (details see Table 1) in this work. The first two data sets [17,18] were chosen for the task of cell type clustering at 1 Mb resolution and were also used by scHiCluster [32], a method mainly designed for cell type clustering. The first one provided three cell types [18] (i.e., Oocyte, Zygote-P, and Zygote-M), and the second one [17] included four cell types (i.e., HeLa, HAP1, GM12878, and K562). 

The second data set was also used for the detection of TADs at 50 kb resolution because the bulk Hi-C data for these cell types were also publicly accessible [2]. Therefore, we can explore the similarities of TADs called from single-cell and bulk Hi-C data. 

The third data set (Li, et al., 2019) contained simultaneously profiled DNA methylation and single-cell Hi-C, which was used to explore the relationship between methylation and TAD boundaries. 

We used haploid 2i-maintained and serum-maintained cells as the last data set [15], which contained single-cell Hi-C data and the cell cycle phase of every single cell. We used this data set to reconstruct 3D genome structures and explored the compactness of the 3D structures in each cell cycle phase. 

For the first two single-cell data sets, we filtered out some single cells with depleted contacts by only keeping the cells that had >5000 non-diagonal contacts at 500 kb resolution. The details about the number of cells we obtained before and after filtering are present in Supplementary Table S1. 

The bulk Hi-C data for HeLa were downloaded using juicer [2]. We called TADs based on the bulk Hi-C data of HeLa using both domaincaller [7] (1156 TADs detected) and TopDom [29] (4812 TADs detected) at 50 kb resolution. A combination of the two TAD sets was considered as the TADs called on the bulk Hi-C data of HeLa.

### 2.2. Overview of the scHiCEmbed Pipeline

**Figure 1:**
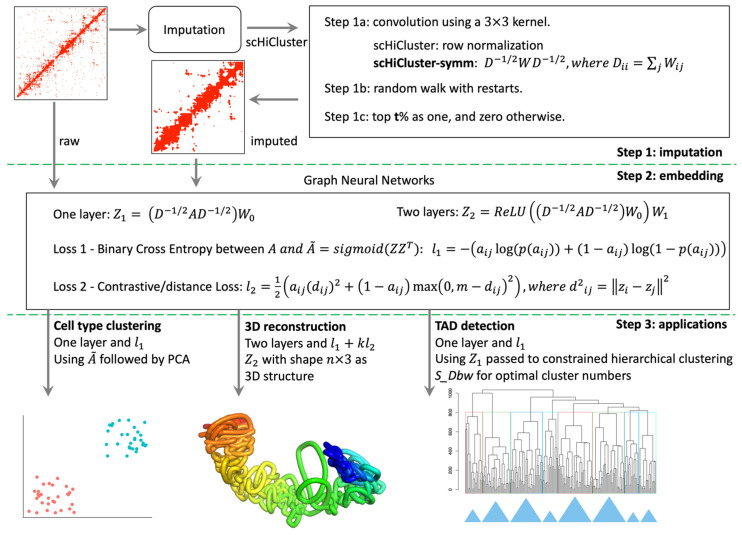
The scHiCEmbed pipeline. It contains three main steps: (1) imputation with revised scHiCluster, (2) embedding with graph auto-encoders, and (3) applications for cell-type identification, 3D structure reconstruction, and TAD detection.

The scHiCEmbed contains three main steps, see Figure 1: (1) single-cell Hi-C imputation using the revised scHiCluster; (2) embed the raw or imputed single-cell Hi-C matrix and obtain the embedding/latent matrix using a graph auto-encoders; and (3) apply the system to three different applications including cell type clustering, 3D genome structure reconstruction, and TAD detection. 

The scHiCEmbed was designed to simultaneously accomplish three tasks using one graph neural network, that is, using the reconstructed adjacency matrix and embedding matrices to (1) identify cell types, (2) reconstruct 3D genome structures, and (3) detect TADs. For reconstructing 3D structures of individual chromosomes, we set the second dimension of the embedding matrix to three and used the contrastive loss to constrain the Euclidean distances between each pair of nodes. For TAD detection, the optimal embedding matrix from a graph auto-encoder was used for constrained hierarchical clustering. In this way, node/bead pairs that were sequentially successive and spatially close to each other were classified into the same cluster. Each cluster was considered as a TAD.

### 2.3. Imputation of Single-Cell Hi-C Contact Matrices

Since we have modified the method of scHiCluster [32] and used its output as our input for graph auto-encoders, here we briefly describe its procedures and our modifications. 

Due to the challenges of whole-genome DNA sequencing in single-cell experimental procedures (e.g., dropouts from material loss), there are always some missing values in single-cell Hi-C contact matrices. Therefore, it is necessary to impute single-cell Hi-C contact matrices before further processing. The first step of imputation in scHiCluster, which is similar to the first step of HiCRep [34] (smoothing the raw Hi-C contact matrix), is to interpolate the missing value in a single-cell Hi-C contact matrix by applying a n×n convolution filter matrix *F*, where n=1+2×h (*h* is the given window size). The raw single-cell Hi-C contact matrix *A* is converted into an imputed matrix *B* using the following formula:

(1) Bij=∑pqFApq∑F

where i−h≤p≤i+h, and j−h≤q≤j+h. The elements in the filter are all set to one. The parameter *h* is set to one in this study. 

The second step of scHiCluster is random-walk-based imputation. The imputed matrix *B* from step 1 is first normalized by row normalization:

(2) $$C_{ij}=\frac{B_{ij}}{\sum_{j^{\prime }}B_{{ij}^{\prime }}}$$

where $j^{\prime }$ is column index ranging from one to the number of columns. 

Random walk with restarts (RWR) is then used to capture the global and local topological structures of the networks represented in matrix *C*. The equation for RWR is

(3) $$D_{t}=pD_{t-1}C+\left(1-p\right)e$$

where $D_{t}$ represents the matrix after the *t*th iteration, *e* denotes the identity matrix, $D_{0}$ equals *e*, and *p* is simply set to 0.5 for equally balancing the global and local structures. The stopping criterion for RWR is $\|D_{t}-D_{t-1}\|{}_{2}\leq 10^{-6}$. 

The third step of scHiCluster is to convert the matrix *D* into a binary matrix *E*. A threshold *t* is set to label the percentile of *D*; if an entry in *D* belongs to the top *t*% of all values in *D*, the corresponding entry in *E* is set to one, and zero otherwise. 

Row normalization in Equation (2) makes the imputed matrix *B* non-symmetric, resulting in that the follow-up three matrices (*C*, *D*, and *E*) are also non-symmetric. It does not matter for the task of cell type clustering, but when we think of matrix *B* as a graph adjacency matrix, in which the entries represent edge weights, *B_ij_* equaling *B_ji_* is more appropriate. To make matrix *B* symmetric, we use the following normalization method instead of row normalization:

(4) $$C=W^{-1/2}BW^{-1/2}$$

where *W* is a diagonal matrix with diagonal entries equal to the sum of the corresponding rows in *B*. This normalization method has also been used in a revised RWR [35] and graph auto-encoders [33]. 

The original scHiCluster consists of the three main steps represented in the three equations (i.e., Equations (1)–(3)). We further defined the revised scHiCluster (scHiCluster-symm) using the three equations (i.e., Equations (1), (3), and (4)), from which the intermediate matrices (*B*, *C*, and *D*) and the final output matrix (*E*) were all symmetric. We will show in the results section that scHiCluster-symm has competitive performance with the original scHiCluster in cell type identification.

### 2.4. Bin-Specific Embeddings Using Graph Auto-Encoders

A graph auto-encoder is an unsupervised framework for learning graph-structured data [33,36]. It usually contains two main parts: the encoder consists of one or more graph convolutional layers for embedding each node into a higher-dimensional space, and the decoder uses the learned embeddings to reconstruct the adjacency matrix of the input graph. 

For a typical graph convolutional layer [36], the encoder operation Z=AXW is performed, where matrix *A* is the n×n adjacency matrix (*n* is the number of nodes in the graph), *X* is the n×m node feature matrix (*m* is the number of features for each node), and *W* is the m×p weight matrix (*p* is the dimensionality). The embedding matrix *Z* is usually followed by a ReLU [37] for non-linearity if the current layer is not the last encoder layer. The decoder is simply $\hat{A}=\sigma \left(ZZ^{T}\right)$, where *σ* is the sigmoid function. 

The limitation of the typical graph convolutional layer is that the node feature matrix is needed as the input. If only the adjacency matrix is available, researchers have simplified the graph convolutional layer to Z=AW [33,38] (replacing *X* with an identity matrix), in which *W* is an n×p weight matrix, indicating that we embed each node in the graph into *p*-dimensional space. 

In this study, we used the abovementioned straightforward graph auto-encoder framework [38]. The encoder is one layer with a simplified linear graph convolutional operation. The decoder is the same as the one we just described. Therefore, the encoder that only contains one layer can be described as

(5) $$Z_{1}=\left(D^{-1/2}AD^{-1/2}\right)W_{0}$$

where the input adjacency matrix *A* is either a raw single-cell Hi-C matrix or a scHiCluster-symm-imputed binary matrix, *D* is a diagonal matrix with (*i*,*i*)-element equal to the sum of the *i*th row of A, and *W*_0_ is the weight matrix we are trying to learn. 

We considered two types of the adjacency matrix *A* in this work: (1) the raw single-cell Hi-C contact matrix (Hi-C contacts larger than zero indicating an existent edge), and (2) the binary matrix *E* from the scHiCluster-symm. If the contacts in a raw matrix are larger than zero or the entry is one in the binary matrix that is generated from scHiCluster-symm, we consider there is an edge between the two beads/bins. 

Based on our testing results, these one-layer graph models can result in a satisfactory performance with most input graphs. However, when we embed bins in a lower-dimensional space (e.g., 3D), the one-layer graph models were hard to train. Therefore, to embed bins more accurately in 3D space for 3D structure reconstruction, we also used a two-layer graph model:

(6) $$Z_{2}=ReLU\left(\left(D^{-1/2}AD^{-1/2}\right)W_{0}\right)W_{1}$$

where the first layer is the same as the previous one-layer model, but it is then followed by a ReLU for nonlinearity and finally, in the second layer, multiplied by a second weight matrix *W*_1_. 

In our two-layer model, the first layer is designed for embedding bins in a higher-dimensional space (e.g., 128), and the second layer is for reducing the dimension to a lower space (e.g., 3). For example, if *W*_0_ is n-by-128 and *W*_1_ is 128-by-3, the final embedding *Z* will be an n-by-3 matrix, which is thought of containing the 3D coordinates of the reconstruction structure. 

We tested two loss functions to learn our weight matrices. The first loss function is binary cross-entropy (BCE) between the input adjacency matrix *A* and the reconstructed adjacency matrix $\tilde{A}=sigmoid\left(ZZ^{T}\right)$. For each entry $a_{ij}$ in the adjacency matrix *A*, the first loss is

(7) $$l_{1}=-\left(a_{ij}\log\left(p\left(a_{ij}\right)\right)+\left(1-a_{ij}\right)\log\left(1-p\left(a_{ij}\right)\right)\right)$$

where $p\left(a_{ij}\right)$ is the probability that there is an edge between the two bins *i* and *j* in the reconstructed adjacency matrix. Therefore, the task of the first loss function is to optimally recover as many positive and negative edges as possible in the reconstructed adjacency matrix.

The second loss function is contrastive loss [39]. For each entry $a_{ij}$ in the adjacency matrix A, the second loss is

(8) $$l_{2}=\frac{1}{2}\left(a_{ij}{\left(d_{ij}\right)}^{2}+\left(1-a_{ij}\right)\max{\left(0,\;m-d_{ij}\right)}^{2}\right)$$

where $d^{2}{}_{ij}={\left\|z_{i}-z_{j}\right\|}^{2}$, the max function is the same as a ReLU, and *m* here is a margin parameter and is simply set to 1 in this work. If there exists a contact between the two bins *i* and *j*, we try to minimize their distance. Otherwise, we make their distance at least larger than or equal to the margin parameter *m*.

### 2.5. Training, Validation, and Blind Test

Positive edges are defined as the edges that are confirmed by the input Hi-C matrix, and the input Hi-C matrix can be a raw Hi-C contact matrix or a scHiCluster-symm-imputed matrix. In other words, the input Hi-C matrix indicates the existence of these positive edges. All positive edges in a graph were randomly split into three subsets based on the ratio 7:2:1 for training, validation, and blind test. 

The negative edges are defined as the edges that do not exist based on the input Hi-C matrix. We randomly selected negative edges for validation and blind test so that the numbers of positive edges and negative edges in the validation and blind test datasets are the same. We implemented one-layer and two-layer graph models using PyTorch [40]. Adam [41] was used as the optimizer. 

The goal of the training process is to successfully reconstruct the input adjacency matrix, that is, recover masked edges. For evaluating the performance of our graph-neural-network models in reconstructing the adjacency matrix, we used two metrics: average precision (AP) and mean area under the receiver operating characteristic (ROC) curve (AUC) (more details about AP and AUC in the supplementary document). The model that achieved the highest validation AP was chosen as the best graph model. The AP and AUC of the best model on testing edges are reported in the results section.

### 2.6. Tuning Hyperparameters

We have two hyperparameters to tune in our 1-layer graph neural network: learning rates and hidden dimensions. We tested two different learning rates (0.01 and 0.001) and four hidden dimensions (16, 32, 64, and 128). Therefore, there are eight combinations of learning rates and hidden dimensions, including (0.001, 16), (0.001, 32), (0.001, 64), (0.001, 128), (0.01, 16), (0.01, 32), (0.01, 64), and (0.01, 128). For each of the eight combinations, we used the corresponding learning rate and hidden dimension to train our graph auto-encoders. The input of our graph neural network is the raw Hi-C contact matrix or the scHiCluster-symm-imputed matrix for each chromosome from data set 1 at 1 Mb resolution with the top *t* parameter in scHiCluster set to 20. 

The test AP and AUC results are shown in Supplementary Figure S1 and Table S2, from which we can observe the following points: (1) the learning rate of 0.01 is a better choice; (2) different hidden dimensions do not significantly affect the performances; and (3) using imputed single-cell Hi-C data as input to scHiCEmbed results in models with better testing performance than directly using raw single-cell Hi-C data. The reason may be that some isolated contacts in raw single-cell Hi-C matrices were removed after imputation. In the downstream analysis, we used the learning rate of 0.01 and the hidden dimension of 128 for all 1-layer graph models. Since our 2-layer graph model is particularly designed for 3D structure reconstruction, the two hidden dimensions for the two weight matrices of the first and second layers are 128 and 3, respectively.

### 2.7. Cell Type Clustering

For each cell, we implemented two-round of dimensionality reductions using principal component analysis (PCA) in three steps: (1) reduced raw, scHiCluster-imputed, or scHiCEmbed-reconstructed single-cell Hi-C matrix for each chromosome to a given dimension, (2) concatenated reduced vectors of all chromosomes from step 1, and (3) reduced the concatenated vectors to 2D space, which was the input of k-means for cell type clustering. We used the adjusted rand index (ARI) to measure the performance of k-means clustering.

### 2.8. 3D Genome Reconstruction

A learned embedding matrix ($Z_{1}$ from Equation (5) or $Z_{2}$ from Equation (6)) is an *n*-by-3 matrix, where *n* stands for the number of beads. We directly treated the learned embedding matrices as the 3D coordinates of the final reconstructed 3D chromosomal structures. Given a raw single-cell Hi-C matrix, we first trained our graph auto-encoder model and then extracted the optimal embedding matrix when the validation AP achieves the best. In this work, we designed and benchmarked three graph models for 3D genome reconstruction: (1) 1-layer linear encoder (Equation (5)) along with BCE loss function ($l_{1}$); (2) 2-layer encoder (Equation (6)) also only with BCE loss function ($l_{1}$); and (3) 2-layer encoder (Equation (6)) with a combination of BCE and contrastive loss functions ($l_{1}+kl_{2}$, *k* is simply set to one).

### 2.9. Calling TADs Based on Embeddings

The embedding matrix *Z* (that we learned from graph neural networks as mentioned in Section 2.4.) can be thought of as the coordinates of the beads/nodes in *p*-dimensional space. Therefore, we used the embeddings to generate a dissimilarity matrix (squared Euclidean distances). After that, we iteratively used constrained hierarchical clustering to merge the two clusters that were sequentially next to each other and have the smallest distance into one new cluster. Finally, we obtained a dendrogram illustrating the structures of hierarchical clusters, in which each cluster was considered a TAD. 

We used the implementation of constrained hierarchical clustering (CONISS) in an R package Rioja [42], which was also used in TADpole [43] for calling TADs based on bulk Hi-C data. After obtaining a dendrogram, we used *S_Dbw* [44] to determine the optimal number of clusters. Each final cluster was taken as a TAD. The work [45] benchmarked 11 widely used clustering validation methods and concluded that *S_Dbw* was the best one, which used cluster variance to measure the compactness of data sets and used the density between clusters to measure separation. 

The overlap coefficient was used to measure the similarities of TAD boundaries called on different single-cell Hi-C data sets, including raw single-cell Hi-C (scHiCEmbed-raw) and scHiCluster-imputed single-cell Hi-C with different top *t* parameters (scHiCEmbed). 

### 2.10. TADs and Methylation

We explored the relationship between TAD boundaries and methylation density on data set 3 at 50 kb resolution. The methylation data for every single cell were downloaded from GEO, GSE119171. Each TAD has two boundaries, and for each boundary, we extended it towards both sides up to 400 kb and calculated the average methylation density for each 10 kb bin.

## 3. Results

### 3.1. Cell Type Clustering

The existing tool scHiCluster was mainly designed for single-cell Hi-C clustering on different cell types. Here we tested the abilities to distinguish different cell types for five types of single-cell Hi-C data: raw, imputed Hi-C data by the original scHiCluster (scHiCluster-imputed), imputed Hi-C data by the scHiCluster that was modified by us (scHiCluter-symm-imputed), adjacency matrices reconstructed by our scHiCEmbed with raw Hi-C as input (scHiCEmbed-raw), and the adjacency matrices reconstructed by our tool scHiCEmbed with scHiCluster-symm-imputed Hi-C data as input (scHiCEmbed) on data sets 1 and 2 at 1Mb resolution. 

**Figure 2:**
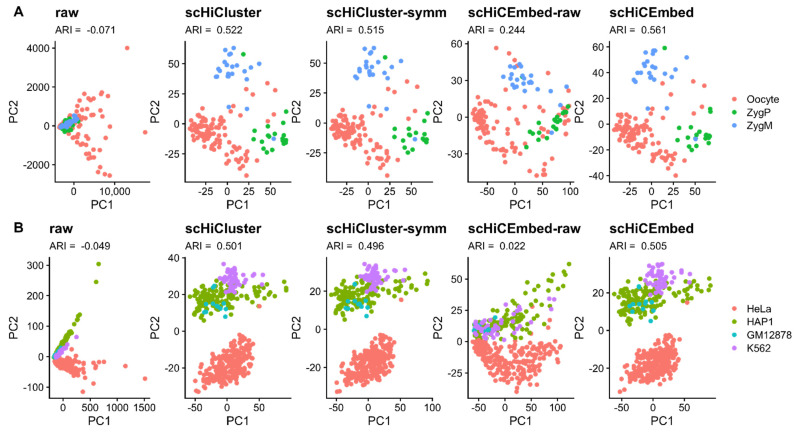
Results for cell type clustering (PC1 vs. PC2) on data sets 1 (**A**) and 2 (**B**) at 1 Mb resolution for raw single-cell Hi-C and imputed Hi-C from four methods, including scHiCluster, scHiCluster-symm, scHiCEmbed-raw, and scHiCEmbed.

The clustering results (PC1 vs. PC2) are shown in Figure 2 (subplot A for data set 1 and subplot B for data set 2). The additional clustering results (PC2 vs. PC3) can be found in Supplementary Figure S2. We used the adjusted rand index (ARI) to measure k-means clustering performance. From Figure 2, we can conclude that our tool scHiCEmbed and our modified scHiCluster (scHiCluster-symm) achieve comparable performance with the original scHiCluster on both data sets, and even scHiCEmbed-raw improves the performance of using raw single-cell Hi-C data. Our competitive results of cell type clustering also indicate that scHiCEmbed can successfully recover adjacency matrices.

### 3.2. 3D Genome Structure Reconstruction

**Figure 3:**
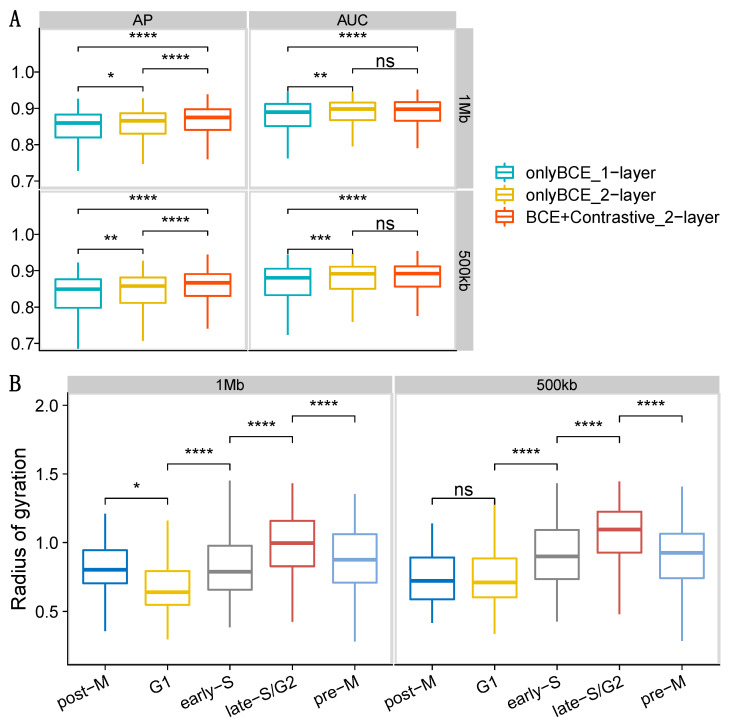
Results for 3D genome reconstruction on data set 4 (haploid 2i-maintained cells) at 1 Mb and 500 kb resolutions for scHiCEmbed with raw single-cell Hi-C as input. (**A**) graph network evaluations using AP and AUC for three different combinations of loss function and number of layers. (**B**) The radius of gyration of our reconstructed 3D genome structures at different cell stages. ns: *p*-value > 0.05, *: *p*-value ≤ 0.05, **: *p*-value ≤ 0.01, ***: *p*-value ≤ 0.001, ****: *p*-value ≤ 0.0001.

We reconstructed 3D structures of the whole genomes on data set 4 with raw genome-wide single-cell Hi-C data as the input at 1 Mb and 500 kb resolutions. Subplot A in Figure 3 shows the AP and AUC results of reconstructing the adjacency matrix for three different networks on haploid 2i-maintained cells (see Supplementary Figure S3 for haploid serum-maintained cells). Here we benchmarked three graph models: one-layer with BCE loss, two-layer with BCE loss, and two-layer with both BCE and contrastive loss functions. Both two-layer models and contrastive loss significantly improved the performance in terms of AP and AUC (Figure 3A and Figure S3A), and all of the three testing models achieved high performance (median AP > 0.85 and median AUC > 0.85).

In Figure 3B we used the radius of gyration (Rg) to measure the compactness of our reconstructed structures. The radius of gyration is the root mean square distance between nodes and their center of mass and has been widely used in measuring the compactness of a 3D structure [10]. Therefore, if a 3D structure is highly compacted, its Rg will be very small. The middle three stages (i.e., G1, early-S, and late-S/G2) belong to the interphase state in the cell cycle. The rising Rg values in Figure 3B and Figure S3B indicate the graduate expansion of our reconstructed chromatin 3D structures in the 3D space, which fits the biological meaning. 

**Figure 4:**
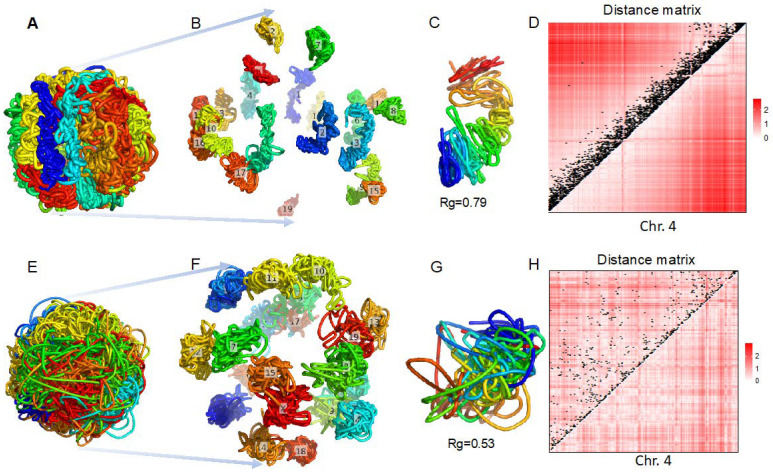
The scHiCEmbed reconstructed 3D genome at 500 kb resolution for one cell in post-M stage (**A**–**D**) and another cell in G1 stage (**E**–**H**). (**A**,**E**) reconstructed 3D structures of whole genomes (chromosomes are labeled in different colors). (**B**,**F**) expand a nucleus into separate chromosomes. (**C**,**G**) reconstructed 3D structures of chromosome 4 with their radius of gyrations. (**D**,**H**) Euclidean distance matrices parsed from reconstructed 3D structures corresponding to (**C**,**G**), respectively. We overlay raw Hi-C contacts on the distance matrices (black points on heat maps (**D**,**H**)).

Two examples of our reconstructed whole-genome 3D structures for a cell in the post-M stage and another cell in the G1 stage at 500 kb resolution are shown in Figure 4. For the reconstructed 3D structures of the whole genomes, individual chromosomes of the cell in the post-M stage have elongated shapes (Figure 4A,B), whereas chromosomes of the cell in the G1 stage intertwined with each other (Figure 4E,F).

For one specific chromosome, that is, chromosome 4, we drew the reconstructed 3D structures (Figure 4C,G), and distances matrices parsed from the reconstructed 3D structures (Figure 4D,H) with Hi-C contacts overlaid. We observed that raw Hi-C contacts in post-M stage were mostly located near the diagonal (Figure 4D), resulting in an elongated 3D structure (Figure 4C) and longer distances in the regions far away from the diagonal (Figure 4D), whereas raw Hi-C contacts in G1 stage were distributed all over the square matrix (Figure 4H), making the reconstructed 3D structure highly compacted (Figure 4G) and longer distances distributed dispersedly (Figure 4H).

**Figure 5:**
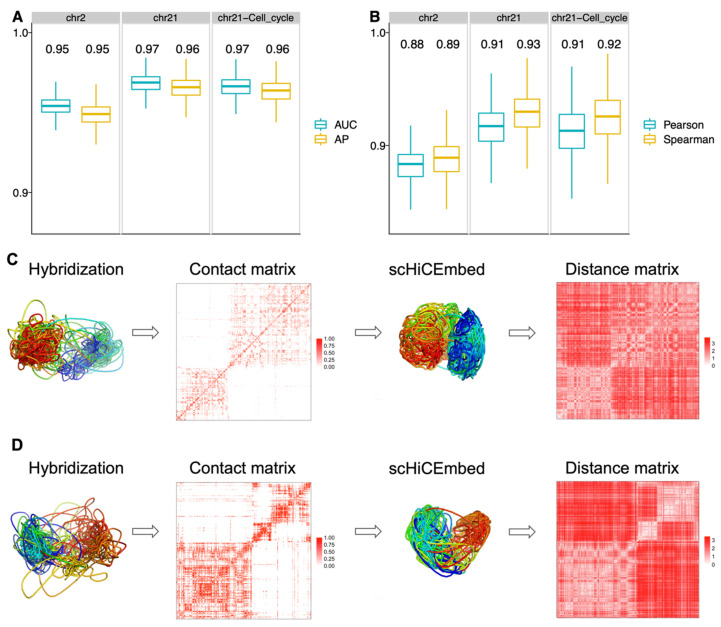
Results for scHiCEmbed-reconstructed 3D structures. (**A**) AP and AUC for the three chromosome sets (chr. 2, chr. 21, and chr. 21 with cell cycle). Mean values are included in each boxplot. (**B**) Pearson correlation coefficients and Spearman’s rank correlation coefficients between scHiCEmbed-recovered Hi-C matrices and distance matrices parsed from our scHiCEmbed-reconstructed 3D structures (absolute values provided). Mean values are included in each boxplot. (**C**,**D**) 3D structures for two different chromosomal copies (#106 of chr. 21 with cell cycle and #183 of chr. 2), from left to right are 3D structures determined by the sequential hybridization approach, binary contact matrices parsed from hybridization-determined structures, scHiCEmbed-reconstructed 3D structures, and distance matrices parsed from scHiCEmbed-reconstructed structures.

We downloaded 15,178 3D structures of individual chromosomes (3029 copies of chromosome 2, 7591 copies of chromosome 21, and 4558 copies of chromosome 21 with cell-cycle markers) determined by the sequential hybridization approach [46]. For each 3D structure, we first generated its distance matrix and then created a binary contact matrix by assigning one for those distances shorter than 500 nm and assigning zero for the rest. This binary contact matrix was passed to our pipeline scHiCEmbed for reconstructing the 3D structure. The scHiCEmbed has achieved very high AP and AUC (Figure 5A, average values ≥ 0.95), indicating that our tool can successfully recover the input binary contact matrices. 

We next calculated Pearson correlation coefficients and Spearman’s rank correlation coefficients between our recovered Hi-C matrices and distance matrices parsed from our reconstructed 3D structures (Figure 5B, average absolute values ≥ 0.88). These high correlation values suggest that our reconstructed 3D structures correctly match our recovered Hi-C matrices, which are remarkably consistent with our input Hi-C matrices based on the almost perfect AP and AUC values. We visualized four chromosomal structure-related entities (hybridization structures, binary contact matrices, scHiCEmbed-reconstructed structures, and distance matrices parsed from our reconstructed structures) for chromosome 2 (Figure 5C, copy #106), chromosome 21 with cell-cycle markers (Figure 5D, copy #183), and the other three chromosomal copies shown in Supplementary Figure S4. Both hybridization and scHiCEmbed-reconstructed 3D structures show two big and separated components (Figure 5C,D), and our parsed distance matrices have similar patterns to the binary contact matrices (Figure 5C,D and Figure S4).

### 3.3. TAD Detection

#### 3.3.1. Detecting Naïve TADs

To evaluate the capability of our scHiCEmbed for detecting TADs, we generated naïve TADs based on TAD definitions on bulk Hi-C of HeLa using TopDom at 50 kb resolution. For each chromosome, we generated a 2D contact matrix at 50 kb resolution; all entries within a TAD were set to one, and zero otherwise. In this way, we not only obtained the input adjacency matrices for scHiCEmbed but knew the locations of TAD boundaries in advance. 

**Figure 6:**
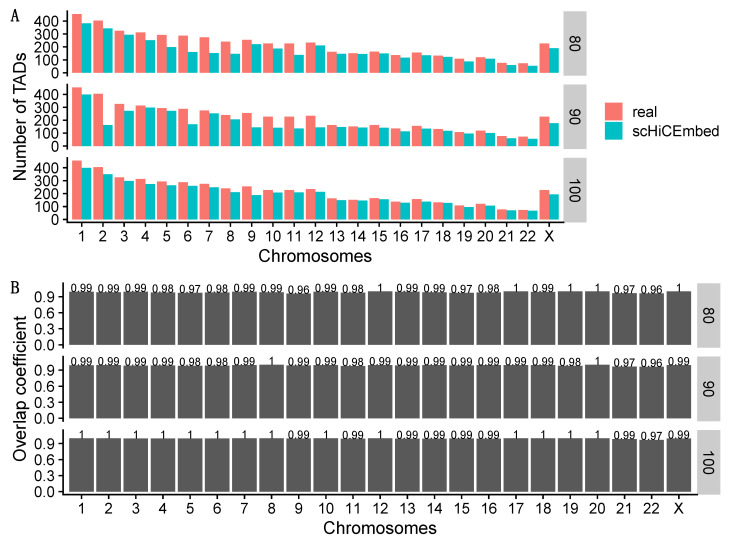
Results for detecting naïve TADs. (**A**) number of TADs in the real adjacency matrix and detected by scHiCEmbed with the three self-defined matrices (100%, 90%, and 80%) as input. (**B**) overlap coefficient between real and scHiCEmbed-detected TAD boundaries with allowing ±1 bin mismatch.

We also generated two more input contact matrices for each chromosome by randomly selecting 10% and 20% entries within each TAD and setting them to zero. The number of TADs that were detected by scHiCEmbed using the three input matrices (100%, 90%, and 80%) and the overlap coefficients between real and scHiCEmbed-detected TAD boundaries are shown in Figure 6. We observed that (1) scHiCEmbed could detect almost all of the TADs based on all of the three input matrices; and (2) the overlap coefficients between the real and scHiCEmbed-detected TAD boundaries were >0.96 on all chromosomes, which indicated that our TAD detection methods could successfully locate the hidden TAD boundaries.

#### 3.3.2. Detecting TADs Based on Raw and Imputed Single-Cell Hi-C Data

**Figure 7:**
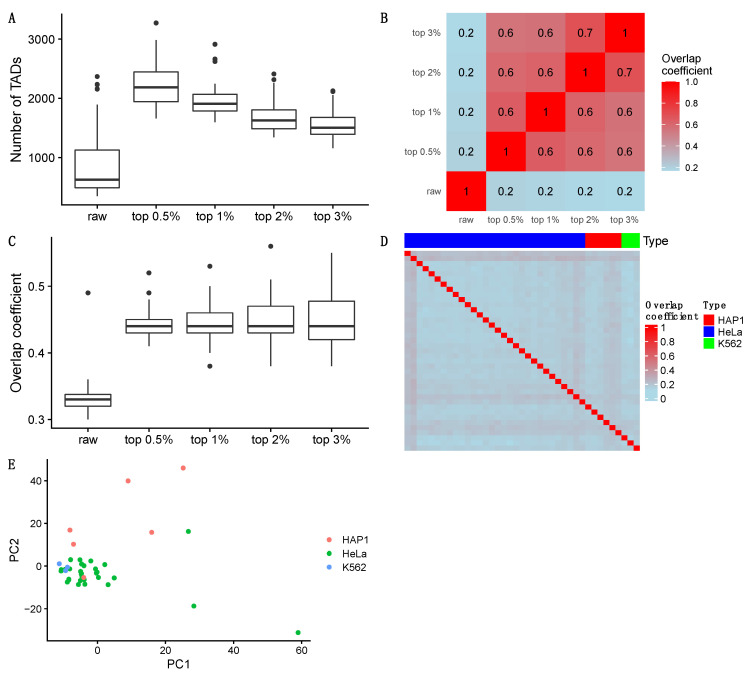
Results for TAD detection on 30 HeLa cells at 50 kb resolution for raw single-cell and imputed (four different top parameters) Hi-C. (**A**) The number of TADs scHiCEmbed detected on raw and imputed (four different top parameters) single-cell Hi-C. (**B**) Average overlap coefficient between each pair of the five TAD-boundary sets when allowing ±1 bin mismatch. (**C**) Overlap coefficients between each of the five TAD boundary sets and TAD boundaries called from bulk Hi-C when allowing ±1 bin mismatch. (**D**) Overlap coefficients between any two TAD boundary sets of 39 single cells (30 HeLa cells, six HAP1 cells, and three K562 cells) called on imputed single-cell Hi-C data with top 1% parameter when allowing ±1 bin mismatch. (**E**) Dimensionality reduction in genome-wide TAD boundary profiles for the 39 single cells.

We selected 30 cells of HeLa that each of them has single-cell contacts larger than or equal to 30,000 on data set 2 for detecting TADs at 50 kb resolution. In Figure 7A, we showed the numbers of TADs that were detected by scHiCEmbed using five different input graphs: a raw single-cell Hi-C matrix and four imputed single-cell Hi-C matrices with different top *t* parameters for scHiCluster-symm. It can be found that with more top contacts incorporated, the number of TADs that were detected keeps reducing. This makes sense because more imputed contacts make the boundaries unclear, resulting in fewer TADs that have larger lengths. 

We compared TAD boundary similarities using the overlap coefficient between each pair of the five data sets in Figure 7B. It can be found that TAD boundaries from imputed Hi-C data with different top percentage contacts share more common boundaries than with raw single-cell Hi-C data. 

Moreover, we compared TAD boundary similarities between using single-cell Hi-C and bulk Hi-C data in Figure 7C. From the results, we find that (1) more than half of the TAD boundaries are not in common between these two Hi-C types; and (2) imputed single-cell Hi-C data share more common boundaries with bulk Hi-C data than raw single-cell Hi-C. 

We collected 39 single cells (30 HeLa cells, six HAP1 cells, and three K562 cells) containing the greatest number of Hi-C contacts within their cell types. The overall small overlap coefficient values between each pair of the 39 cells shown in Figure 7D indicate that TAD boundaries are cell-specific. Specifically, individual cells with the same or different cell types have significantly different TAD boundaries even though they have similar Hi-C embeddings, which is consistent with previous works [47,48]. However, we observed that there were seven individual cells (four HeLa cells and three HAP1 cells) sharing more boundaries with most of the total 39 single cells. These seven single cells may share some common TAD patterns with almost all the other cells although these common TAD patterns are not shared between the other cells. 

Next, we used genome-wide TAD boundary profiles to do cell-type clustering of the 39 single cells. TAD boundary profiles/vectors for every single cell were generated by assigning one to boundary bins and assigning zero to the other bins within TADs. The dimensionality reduction results (PC1 vs. PC2) shown in Figure 7E suggest that not all single cells with the same cell types have the similar boundary profiles, but most single cells with different cell types (27 HeLa cells, one HAP1 cell, and all three K562 cells) have relatively similar principal components.

**Figure 8:**
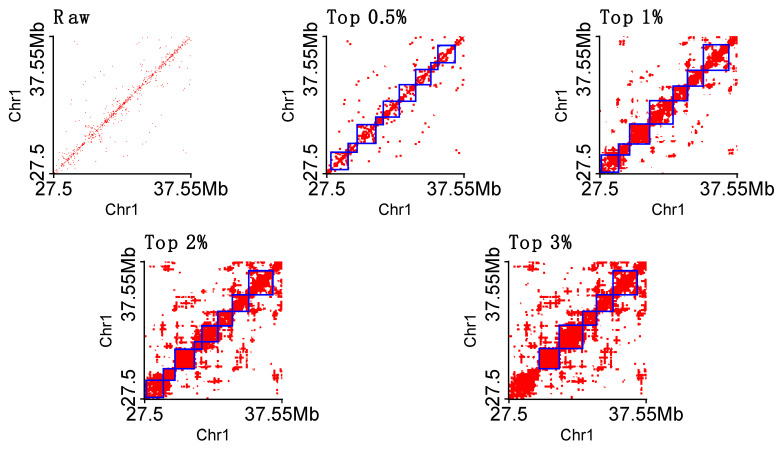
TAD examples at 50 kb resolution called on raw and imputed (four different top *t* parameters) single-cell Hi-C. The blue lines highlight TADs.

In Figure 8, we show an example of raw and imputed Hi-C heat maps that are integrated with detected TADs highlighted by blue lines. It can be found that no TAD is detected on raw single-cell Hi-C data, and the average length of TADs increases when we use more imputed contacts. From this example, we may conclude that the top *t* parameter of 1% may be a better choice at the resolution of 50 kb.

#### 3.3.3. Methylation Loss around the TAD Boundaries

**Figure 9:**
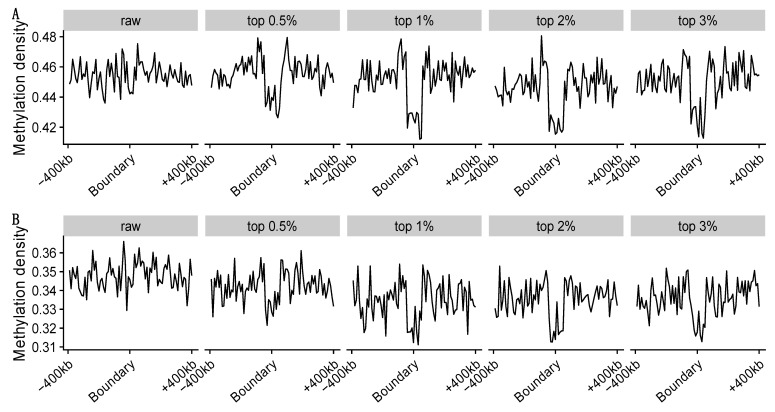
Methylation is depleted around TAD boundaries on data set 3 at 50 kb resolution for two single cells (**A**,**B**). TADs are called on raw and imputed (four different top parameters) single-cell Hi-C.

We further explored the methylation levels around the TAD boundaries of two selected single cells in Figure 9. We observed methylation loss around the TAD boundaries for imputed Hi-C data, but not for the raw single-cell Hi-C data. Researchers have found that TAD boundaries were enriched with CTCF [7] and that CTCF binding sites were usually along with DNA methylation loss [49]. Therefore, the observation of methylation loss around TAD boundaries indicates that the TADs detected by our scHiCEmbed make sense.

## 4. Conclusions

In this study, we developed a bin-specific embedding tool named scHiCEmbed to embed single-cell Hi-C contact matrices using graph auto-encoders. We explored three applications of scHiCEmbed, including cell type clustering, 3D genome reconstruction, and TAD detection. For cell type clustering, our reconstructed adjacency matrices can be used to classify cell types, and the performance is comparable to or slightly better than scHiCluster. For 3D genome structure reconstruction, the embedding matrix that is also the output of the encoders is directly considered as the 3D coordinates of the reconstructed 3D structure. Our two-layer graph model with a combination of BCE and contrastive loss functions achieves the best performance (both AP and AUC > 0.85) compared with the one-layer graph model with only BCE loss function. Our reconstructed 3D structures indicate that chromatins keep expanding in 3D space in the interphase state. The scHiCEmbed achieved almost perfect performances (both AP and AUC ≥ 0.95) in reconstructing the chromosomal structures determined by the sequential hybridization approach. For TAD detection, we used naïve matrices to show the efficiency of scHiCEmbed in locating TAD boundaries. We have further observed that the TADs detected from single-cell Hi-C (raw or imputed) are different from those detected from bulk Hi-C, confirming that single-cell Hi-C data can reveal cell-to-cell variances. The source code for scHiCEmbed is publicly available at http://dna.cs.miami.edu/scHiCEmbed/ accessed on 8 May 2022.

## Figures and Tables

**Table 1:** Single-cell Hi-C data sets used in scHiCEmbed for the three main tasks, including cell type clustering, TAD detection, and 3D genome reconstruction at different resolutions.

Index	References	Cell Clustering	TAD Detection	3D Reconstruction	Resolutions	Cell Types
1	[18]	✓			1 Mb	Human Oocyte, Zygote-P, and Zygote-M
2	[17]	✓	✓		1 Mb and 50 kb	Human HeLa, HAP1, GM12878, and K562
3	[19]		✓		50 kb	Mouse Embryonic stem
4	[15]			✓	1 Mb and 500 kb	Mouse Embryonic stem

## Data Availability

Not applicable.

## Supplementary Materials

The following supporting information can be downloaded at: https://www.mdpi.com/article/10.3390/genes13061048/s1, Figure S1: tuning hyperparameters (learning rates and hidden dimensions) for 1-layer graph neural networks using average precision (AP) and mean Area Under the Receiver Operating Characteristic (ROC) curve (AUC). Two different single-cell Hi-C data sets (raw and scHiCluster-symm-imputed) from data set 1 are used as input to scHiCEmbed. **** indicates *p*-value < 2.2 × 10^−16^ with Student’s *t*-test. Mean values are added above each boxplot; Figure S2: results for cell type clustering (PC2 vs. PC3) on data sets 1 (A) and 2 (B) at 1 Mb resolution for raw single-cell Hi-C and imputed Hi-C from four methods, including scHiCluster, scHiCluster-symm, scHiCEmbed-raw, and scHiCEmbed; Figure S3. Results for 3D genome reconstruction on data set 4 (haploid serum-maintained cells) at 1 Mb and 500 kb resolutions for scHiCEmbed with raw single-cell Hi-C as input. (A) graph network evaluations using AP and AUC for three different combinations of loss function and number of layers. (B) Radius of gyration of our reconstructed 3D genome structures at different cell stages; Figure S4. Results for scHiCEmbed-reconstructed 3D structures of three chromosomal copies. For each of the three rows, from left to right are hybridization-determined 3D structures, binary contact matrices parsed from hybridization-determined structures, scHiCEmbed-reconstructed 3D structures, and distance matrices parsed from scHiCEmbed-reconstructed structures; Table S1: number of cells on data sets 1 and 2 before and after filtering; Table S2. Results of hyperparameter tuning: the median values of AP and AUC for eight different combinations of learning rates and hidden dimensions. Best AP scores are highlighted.
