# Supplementary material for: scHiCEmbed: Bin-Specific Embeddings of Single-Cell Hi-C Data Using Graph Auto-Encoders
Source: Genes (Basel). 2022 Jun 11;13(6):1048. doi: 10.3390/genes13061048 (PMC9222580; doi:10.3390/genes13061048)
Supplement: Supplementary file 1 [file genes-13-01048-s001.zip › genes-1739921-supplementary.pdf]

scHiCEmbed: bin-specific embeddings of single-cell Hi-C data using graph auto-encoders

Supplementary Materials

Details about AP and AUC

We used the two functions (average_precision_score and roc_auc_score) implemented in the scikit-learn library with default parameters for calculating average precision (AP) and mean Area Under the Receiver Operating Characteristic (ROC) curve (AUC), respectively. AP is usually used to indicate the ability of a model in identifying positives among too many negatives and is calculated as

$$AP = \sum_n (R_n - R_{n-1}) P_n,$$

where R_n and P_n are the recall and precision associated with the nth decision threshold. Therefore, AP is an ideal metric when we deal with imbalanced data, such as graph-structured data with a smaller number of edges. AUC is calculated as the area under ROC, which is a very popular metric for evaluating classification models. An AUC of 0.5 indicates a random model, whereas an AUC larger than or equal to 0.8 indicates that the classification model performs excellently.

Table S1. The number of cells on data sets 1 and 2 before and after filtering.

Cell types	# of cells (before filtering)	# of cells (after filtering)
	Data set 1	
Oocyte (NSN and SN)	114	104
ZygP	24	20
ZygM	31	24
Total	169	148
	Data set 2	
HeLa	269	230
HAP1	254	142
GM12878	582	12
K562	326	50
Total	1431	434

Table S2. Results of hyperparameter tuning: the median values of AP and AUC for eight different combinations of learning rates and hidden dimensions. Best AP scores are highlighted.

Learning rate	Hidden dimension	AP		AUC	
		raw	imputed	raw	imputed
0.001	128	0.895	0.965	0.878	0.967
	16	0.863	0.929	0.855	0.935
	32	0.88	0.945	0.867	0.948
	64	0.89	0.956	0.875	0.959
0.01	128	**0.894**	**0.979**	0.871	0.978
	16	0.884	0.977	0.865	0.978
	32	0.888	0.979	0.866	0.979
	64	0.891	0.979	0.869	0.979

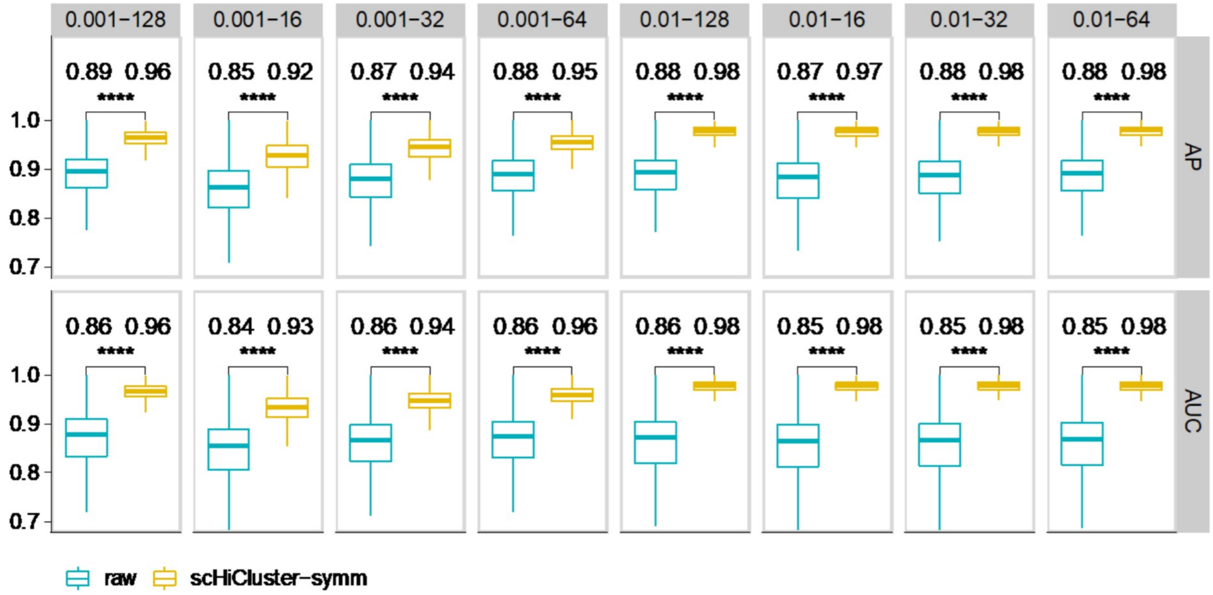

Figure S1. Tuning hyperparameters (learning rates and hidden dimensions) for 1-layer graph neural networks using average precision (AP) and mean Area Under the Receiver Operating Characteristic (ROC) curve (AUC). Two different single-cell Hi-C data sets (raw and scHiCluster-symm-imputed) from data set 1 are used as input to scHiCEmbed. **** indicates p-value < 2.2e-16 with Student's t-test. Mean values are added for each boxplot.

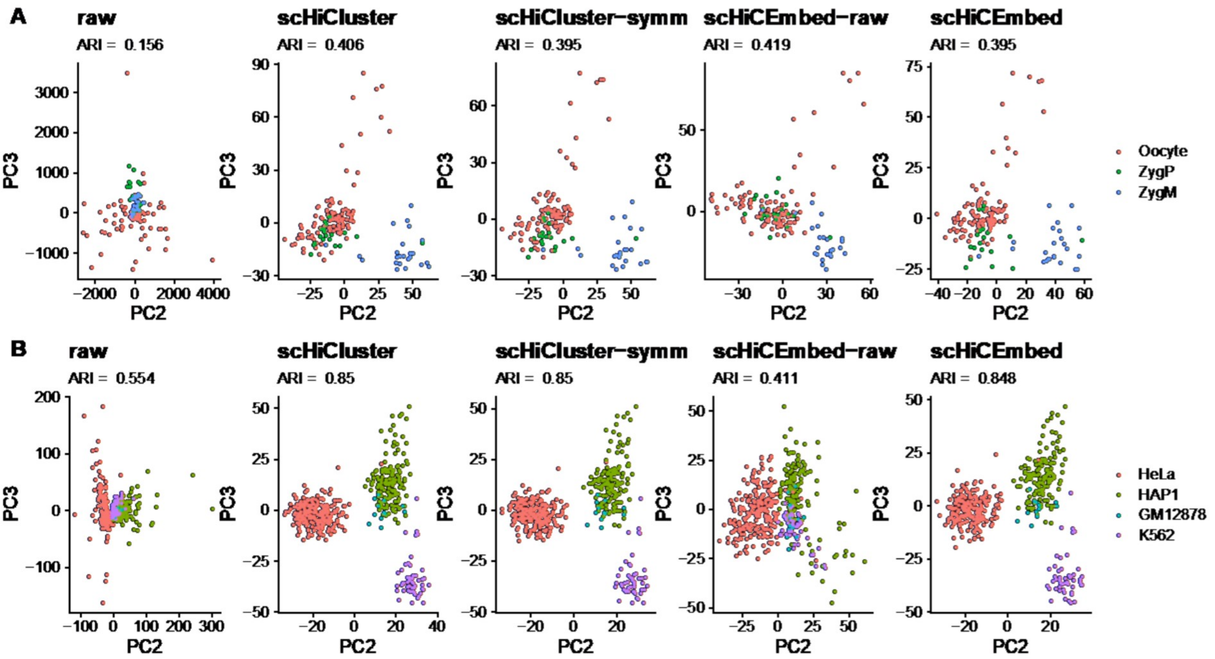

Figure S2. Results for cell type clustering (PC2 vs PC3) on data sets 1 (A) and 2 (B) at 1 Mb resolution for raw single-cell Hi-C and imputed Hi-C from four methods, including scHiCluster, scHiCluster-symm, scHiCEmbed-raw, and scHiCEmbed.

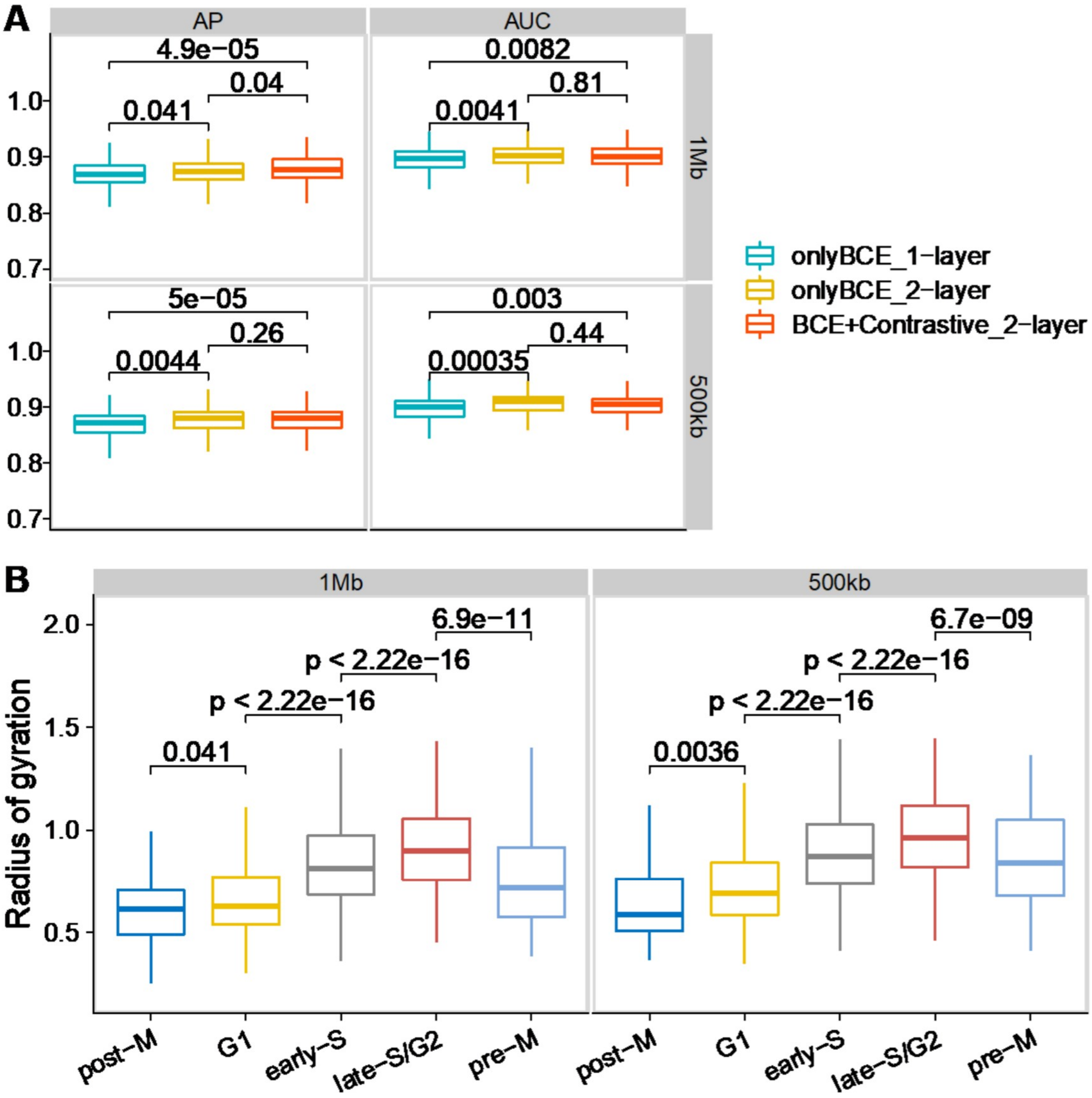

Figure S3. Results for 3D genome reconstruction on data set 4 (haploid serum-maintained cells) at 1 Mb and 500 kb resolutions for scHiCEmbed with raw single-cell Hi-C as input. (A) graph network evaluations using AP and AUC for three different combinations of loss function and number of layers. (B) Radius of gyration of our reconstructed 3D genome structures at different cell stages.

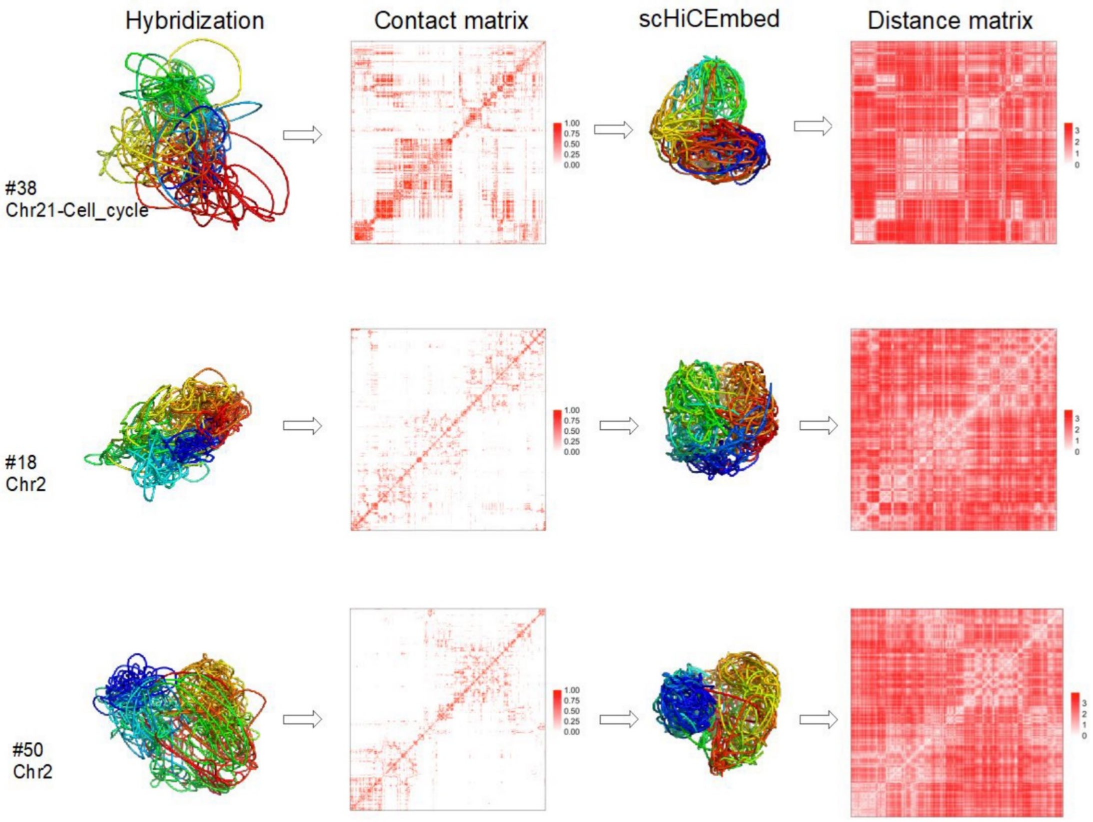

Figure S4. Results for scHiCEmbed-reconstructed 3D structures of three chromosomal sets. For each of the three rows, from left to right are hybridization-determined 3D structures, binary contact matrices parsed from hybridization-determined structures, scHiCEmbed-reconstructed 3D structures, and distance matrices parsed from scHiCEmbed-reconstructed reconstructed structures.
